# Impact of Lung Metastasis versus Metastasis of Bone, Brain, or Liver on Overall Survival and Thyroid Cancer-Specific Survival of Thyroid Cancer Patients: A Population-Based Study

**DOI:** 10.3390/cancers14133133

**Published:** 2022-06-26

**Authors:** Miaochun Zhong, Farhana Zerin Khan, Xianghong He, Lingfei Cui, Kefeng Lei, Minghua Ge

**Affiliations:** 1 Department of Clinical Medicine, Medical College of Soochow University, Suzhou 215006, China; zhongmiaochun@hmc.edu.cn; 2 Department of General Surgery, Cancer Center, Division of Breast Surgery, Zhejiang Provincial People’s Hospital, Affiliated People’s Hospital, Hangzhou Medical College, Hangzhou 310014, China; 3 Department of Pediatric Surgery, Square Hospital Pvt. Ltd., Dhaka 1205, Bangladesh; islam@zju.edu.cn; 4 Public Basic Courses Department, Guangdong University of Science and Technology, Dongguan 523083, China; misshe@163.com; 5 Department of General Surgery, The 7th Affiliated Hospital of Sun Yat-Sen University, Shenzhen 518107, China; cuilf3@mail.sysu.edu.cn; 6 Department of Head and Neck & Thyroid Surgery, Zhejiang Provincial People’s Hospital, Affiliated People’s Hospital, Hangzhou Medical College, Hangzhou 310014, China

**Keywords:** thyroid cancer, lung metastasis, thyroid cancer-specific survival (TCSS), overall survival (OS), surgery

## Abstract

**Simple Summary:**

Patients with thyroid cancer have different survival rate based on their cancer type and the organs affected by the metastases. About 88% of the patients with follicular thyroid cancer are still alive 15 years after diagnosis. Still, some patients will develop metastasis, and the location of the metastases could indicate patients with more aggressive disease.

**Abstract:**

We investigate the impact of lung metastasis versus metastasis of bone, brain, or liver on overall survival (OS) and thyroid cancer-specific survival (TCSS) in patients with thyroid cancer (TC). Therefore, de-identified SEER 18 registry data of primary TC patients diagnosed between 2010 and 2016 were analyzed. The primary outcome was the prognosis of TC patients with lung metastasis compared with other sites. The secondary outcomes included the prognosis comparison between patients with and without surgery and between single and multiple metastasis sites. Isolated lung metastasis was associated with worse OS and TCSS than bone metastasis (both *p* < 0.05) and was associated with worse OS than liver metastasis (*p* = 0.0467). Surgery performed either for the primary or distant site was associated with better OS and TCSS in patients with metastasis of lung or bone (*p* < 0.05). Isolated lung metastasis was related to better OS and TCSS than lung–liver, lung–brain, and lung–other multiple metastases. The multivariable analysis revealed that age < 55 years, surgery to the primary site, and to the distant site(s) were associated with better outcomes, while T4 and Tx were associated with worse outcomes. Nevertheless, it revealed that the other race (i.e., any race other than white, black, or unknown) and male gender were associated with better TCSS only (*p* < 0.05). Isolated lung metastasis is associated with a worse prognosis in TC patients compared with bone or liver metastasis. Surgery performed either for the primary or distant site(s) is associated with better survival outcomes in TC patients with metastasis of lung or bone.

## 1. Introduction

Noncommunicable diseases, often referred to as NCDs, are rapidly becoming the leading cause of death all over the world. Among NCDs, cancer is the top cause of death and a major hurdle to extending life expectancy in practically every country of the world. In 2014, the World Health Organization (WHO) reported that, within the following two decades, the annual incidence of cancer will rise to 22 million, with cancer deaths rising from an expected 8.2 million in 2012 to 13 million in 2032. In 2020, each of the following common types of cancer (lung, female breast, and colorectal) accounted for about 10–12% of all new cases [1,2].

However, thyroid cancer (TC) is one of the most common types of endocrine cancer [3], with the differentiated papillary subtype and follicular subtype being more common than the poorly differentiated subtype and anaplastic subtype [4,5,6]. TC represented an estimated 586,000 new cases in 2020 (ranking in 9th place for incidence) and 44,000 deaths [5]. The management of TC is multidisciplinary and includes surgery, chemotherapy, and radiation therapy [7]. The 15-year OS and TCSS rates of the patients with papillary TC (the most common subtype) are 90% and 99%, respectively [8]. The 15-year TCSS for follicular TC (the second most common subtype) is 88.2% [9].

Although TC generally has a good prognosis, a subset of patients develop metastasis [10,11]. Metastasis is eventually found in about 5% of patients with TC [12]. The main locations of metastasis are the lung and bones [10,11,13,14,15]. Less common sites include the liver, brain, skin, ovaries, and adrenal glands [16,17,18,19,20]. A primary tumor >20 mm is the main risk factor for distant metastasis [21]. The exact prognostic impact of TC metastasis at various sites is unknown in TC. 

Surprisingly, patients with TC and lung metastasis only appear to have a favorable long-term survival [14,15,22], but data from a large cohort with follow-up data are needed to confirm such results. Small-scale population-based data are insufficient because of the low incidence and favorable prognosis of site-specific metastases of TC. 

The Surveillance, Epidemiology, and End Results Program (SEER) database could be helpful in examining the prognosis of patients with metastatic TC because of its huge patient group, relatively complete dataset, and follow-up data. Therefore, this study aimed to predict the prognosis and survival of patients with metastatic TC registered in the SEER database, with a special focus on the incidence and prognosis of lung metastasis. The findings may shed light on the management of metastatic TC.

## 2. Materials and Methods

### 2.1. Study Design and Population

This study was a population-based study based on the SEER database (https://seer.cancer.gov/ (accessed on 24 November 2020)) (The data are obtained from Incidence-SEER 18 Regs Research Data + Hurricane Katrina Impacted Louisiana Cases, November 2018 Sub (1975–2016 varying) in SEER database). This observational study evaluated the de-identified data from the SEER 18 registry by the SEER*Stat 8.3.8 software. The SEER Program is one of the largest databases which contains cancer registration information. The Program is supported by the National Cancer Institute of the United States. The 1975–2016 SEER Research Incidence data (Version submitted in November 2018) mentioned in this article became available in April 2019.

Research on the de-identified data from the SEER program was exempt from the need for institutional review board (IRB) approval by convention, and informed consent was not required. All procedures performed in human subject studies were conducted in conformity with the Helsinki declaration of 1964 and its subsequent amendments or comparable ethical standards.

**Figure 1:**
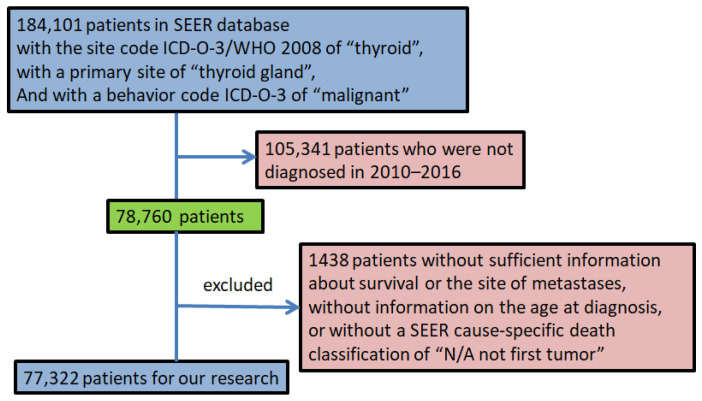
The workflow of patient selection process from the SEER database.

Selection criteria for this study included the following: (1) with primary TC; (2) diagnosed between 2010 and 2016. Patients lacking sufficient information on survival or metastases site, without information on the age at diagnosis, or without a SEER cause-specific death, were excluded from the study. Figure 1 shows the process of selecting patients. 

### 2.2. Data Collection

Data were extracted from the SEER database included race, sex, age at diagnosis, year at diagnosis, marital status, grade, histological type ICD-O-3 [23], T stage, number of positive regional nodes, N stage, the site of metastases, surgery of the primary, surgery of the distant lymph nodes (LNs) or other metastatic sites, SEER cause-specific death classification, vital status, and survival time (months). In the current analysis, TCSS was defined as the time from diagnosis to death from TC. Due to the limitation of data composition in the SEER database in different years, the T staging data were based on a combination of derived AJCC T, 7th ed (2010–2015) data, and derived SEER combined T data. The cases diagnosed between 2010 and 2015 were selected from derived AJCC T, 7th ed (2010–2015) data, and those diagnosed in 2016 were extracted from derived SEER combined T data. It was also used on data from N stages and stage groups. Information about distant LN metastasis was not included for analysis since no data were available on distant LN metastasis in the “Mets at DX-remote LN” variable when diagnoses were made between 2010 and 2015. 

### 2.3. Outcomes

The primary outcome was the prognosis of TC patients with isolated metastasis of the lung compared with those with isolated metastasis of bone, brain, or liver. The secondary outcomes included the prognosis comparison between TC patients who with and without metastasis, with and without surgery on the primary or distant sites, and with metastasis of single site and multiple sites, respectively. 

### 2.4. Statistical Analysis

The chi-square test was used to analyze the demographic and clinical features of thyroid patients with different metastatic sites. The Kaplan–Meier method and log-rank test were used for survival comparisons between patients with lung metastasis, liver metastasis, bone metastasis, brain metastasis, and multiorgan metastases. The Cox proportional hazards model was applied to explore the potential factors associated with the prognosis of patients with metastatic TCS at different sites. *p* values were defined as statistically significant at <0.05. SAS 9.4 (SAS Institute, Cary, NY, USA) was used as the statistical analysis tool.

## 3. Results

### 3.1. Characteristics of Participants

This study comprised 77,322 patients who were diagnosed with TC between 2010 and 2016 and had information about distant metastases. We summarized the demographic and clinical features for all included patients with the different metastatic sites (Table 1 for lung metastasis, Table S1 for other metastases).

Among the 77,322 patients, 998 (1.3%) were diagnosed with lung metastasis, followed by 514 (0.7%) with bone metastasis, 152 (0.2%) with liver metastasis, and 87 (0.1%) with brain metastasis. The patients were then divided according to the number of metastases they had; 1037 (1.3%) patients had single-organ metastasis (711 with isolated lung metastases, 254 with isolated bone metastases, 49 with isolated liver metastases, and 23 with isolated brain metastases), while 326 (0.4%) had multi-organ metastases. Surgical resection of the primary lesion was performed in 543 (54.4%) patients, while surgery of the distant LN(s) or other metastatic site(s) was performed in 118 (11.8%) patients when lung metastases occurred.

### 3.2. Primary Outcomes

**Figure 2:**
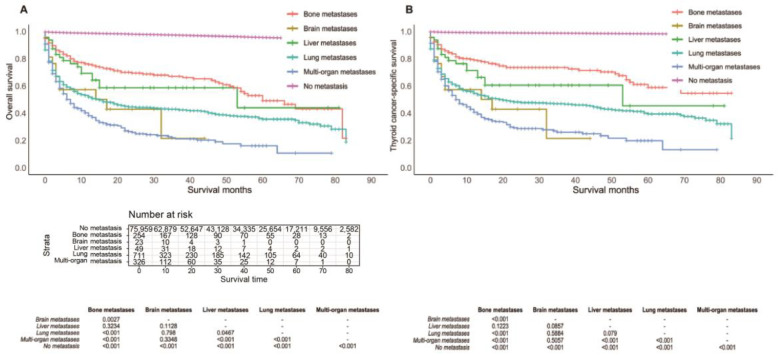
Kaplan–Meier curve of overall survival (**A**) and thyroid cancer-specific survival (**B**) according to the site of metastasis.

The OS and TCSS were compared by pair according to the site of metastasis (Figure 2). Isolated lung metastasis was associated with worse OS and TCSS than isolated bone metastasis (*p* < 0.0001) in patients with TC. There was a slight difference in OS between patients with lung metastasis and those with liver metastasis (*p* = 0.0467) (Figure 2A), but there was no significant difference in TCSS (lung vs. liver metastases: *p* = 0.0790) (Figure 2B). For both endpoints, there were no significant differences between patients with lung metastasis and those with brain metastasis (both *p* > 0.05). Regardless of the metastatic sites of the lung, live, bone, or brain, isolated metastasis was related to better OS and TCSS than multiple metastases.

### 3.3. Secondary Outcomes

The Cox regression analysis results of isolated lung metastasis shown in Table 2 revealed that patient age < 55 years, surgery for the primary site and for the distant site(s) were all associated with better OS and TCSS (*p* < 0.0001, HR = 0.5205, 95%CI: 0.3845–0.7045, patient age < 55 years versus patient age ≥55 years for OS; *p* = 0.0003, HR = 0.5645, 95%CI: 0.4126–0.7723, patient age < 55 years versus patient age ≥ 55 years for TCSS; *p* < 0.0001, HR = 0.2273, 95%CI: 0.1779–0.2904, surgery to the primary site yes versus no for OS; *p* < 0.0001, HR = 0.2294, 95%CI: 0.1776–0.2963, surgery to the primary site yes versus no for TCSS; *p* = 0.0086, HR = 0.5713, 95%CI: 0.3763–0.8674, surgery to the distant site(s) yes versus no for OS; *p* = 0.0061, HR = 0.5304, 95%CI: 0.3371–0.8347, surgery to the distant site(s) yes versus no for TCSS). It also revealed that T4 and Tx were associated with worse OS and TCSS compared with T1 (*p* = 0.0001, HR = 5.0814, 95%CI: 2.2417–11.5184, for OS; *p* = 0.0001, HR = 7.1042, 95%CI: 2.6239–19.2344, for TCSS; *p* = 0.0322, HR = 2.5526, 95%CI: 1.0826–6.0185, for OS; *p* = 0.0207, HR = 3.3817 95%CI: 1.2042–9.4965, for TCSS). Nevertheless, it only revealed the other race and males were associated with the better TCSS (*p* = 0.0298, HR = 0.7166, 95%CI: 0.5305–0.9580, the other race versus white for TCSS; *p* = 0.0338, HR = 0.7944 95%CI: 0.6424–0.9825, male versus female for TCSS).

**Figure 3:**
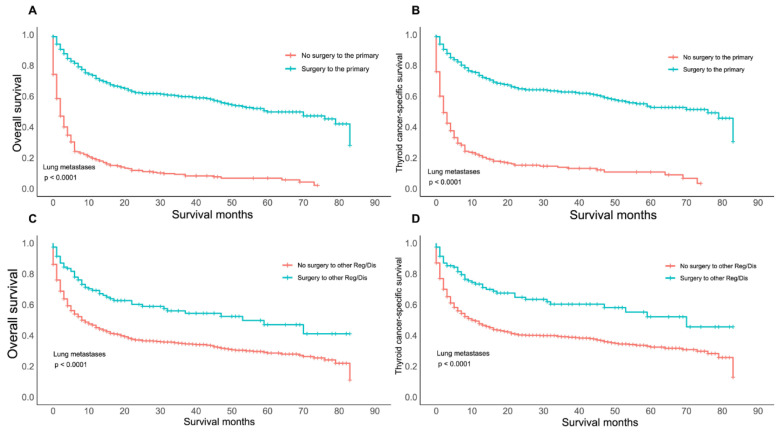
Kaplan–Meier curves of overall survival and thyroid cancer-specific survival according to whether or not surgery of the primary TC lesion and distant metastatic lymph nodes (LN) or lung metastasis was performed. (**A**) Overall survival for patients with isolated lung metastasis with or without surgery of the primary tumor. (**B**) Thyroid cancer-specific survival for patients with isolated lung metastasis with or without surgery of the primary tumor. (**C**) Overall survival for patients with isolated lung metastasis with or without surgery of the distant lymph nodes (LNs) or other metastatic sites. (**D**) Thyroid cancer-specific survival for patients with isolated lung metastasis with or without surgery of the distant lymph nodes (LNs) or other metastatic sites.

Hierarchical analysis revealed that there was a better outcome in patients with lung metastasis if surgery was performed either for the primary or distant site(s) (*p* < 0.05) (Figure 3). Patients with bone metastasis who underwent surgery on primary lesions showed the same survival advantage (*p* < 0.05) (Figure S1). There was no significant difference between the outcomes of patients with liver metastasis (Figure S2) or the OS of those with brain metastasis (Figure S3) with and without surgery of the distant site(s) (for the OS of liver metastasis: surgery vs. no surgery: *p* = 0.1300; for the TCSS of liver metastasis: surgery vs. no surgery: *p* = 0.1900; and for the OS of brain metastasis: surgery vs. no surgery: *p* = 0.05).

**Figure 4:**
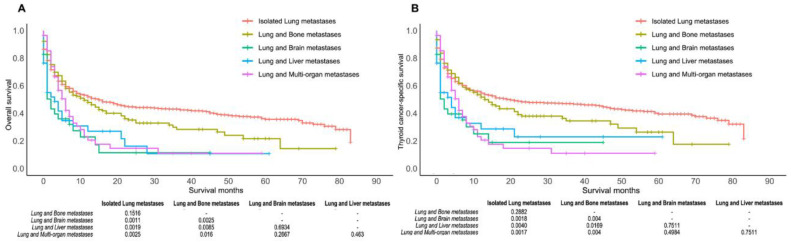
Kaplan–Meier curve of overall survival (**A**) and thyroid cancer-specific survival (**B**) according to the site of metastasis (isolated lung metastasis versus metastasis of ≥2 sites (including lung)).

The result of the comparison of the survival of patients with isolated lung metastasis and multiple-organ metastases, including lung metastasis, is shown in Figure 4. There were significant differences in the prognosis of patients with isolated lung metastasis and those with lung–liver metastases, lung–brain metastases, or lung–other multiple metastases (all *p* < 0.05). In contrast, the OS and TCSS were not different in patients with isolated lung metastasis and those with combined lung–bone metastases (all *p* > 0.05). Similarly, there were no significant differences between the prognosis of combined lung–liver metastases, lung–brain metastases, and metastases of more than three organs, including the lung (all *p* > 0.05).

## 4. Discussion

The main finding of this study was that isolated lung metastasis is associated with a worse prognosis for metastatic TC patients compared with isolated bone or liver metastasis. The results will help generate a better-informed discussion with the patients concerned and help to understand the overall prospect of the disease. Moreover, survival data about whether surgical treatment of the primary or distant LN(s) or other metastatic site(s) is beneficial for TC with lung metastasis and other organ metastases. It will help us to make more appropriate clinical decisions about TC. The results of this study suggested that surgery for primary and metastatic lesions tended to be associated with a better prognosis. In clinical decisions, we should be more active in striving for and even creating surgical opportunities when patients can tolerate them. Surgery is still valuable, even for stage IV metastatic thyroid cancer.

In this study, we found that the survival of patients with lung metastasis from TC was worse than that of those with bone metastasis. In many kinds of cancer, bone metastasis generally has a relatively indolent course and good prognosis [24,25,26], but it was not the same for lung metastasis. Fortunately, as reported by some authors, pulmonary metastasis from TC may also have a good prognosis [22,27]. In this study, there were no differences in survival between lung and brain metastases. A recent study revealed that TC patients who suffered brain metastasis had worse survival than those who suffered lung metastasis [28]. Discrepancies among studies could be due to many factors, including patient selection and sample size.

In this study, the prognostic advantage of surgery over a conservative approach for the primary site of TC was obvious among those with lung and other-organ metastases. Different TC subtypes appear to have different prognoses. Papillary and follicular adenocarcinoma had a better prognosis than papillary adenocarcinoma, which is supported by the literature [29,30,31]. On the other hand, undifferentiated carcinoma had a worse prognosis than papillary carcinoma, also supported by the several reports [32,33]. Nevertheless, for the treatment of metastatic sites, different strategies should be adopted for different locations of metastasis from TC. This study showed that patients with lung and bone metastases with a good prognosis could benefit from surgical treatment of distant LN(s) or other metastatic site(s) in terms of both OS and TCSS. It is similar to the general strategy for other metastatic cancer with a good prognosis, that is, surgery for distant lesions might improve the prognosis [34,35]. An important landmark publication is the 1997 report from Europe and North America, which indicated that the fewer the metastases and the longer the interval before their appearance, the longer survival is after metastasectomy [34,35]. It implied that tumors with a relatively good prognosis might have a longer postoperative survival for metastasectomy [36,37]. It may be an encouraging inference for surgical management. Still, many patients did not undergo surgery of the primary tumor in the group of patients with lung metastases. Indeed, thyroidectomy allows us to deliver radioiodine treatment, which is a milestone in the management of differentiated TC. This study found that 45.5% of patients with distant metastasis did not receive the intervention of the primary lesion, which might be attributed to the high proportion of patients with metastasis complicated with T4 (56.1%), which might lead to a high proportion of primary lesions being inoperable or difficult operations. Still, the SEER database does not record the reasons why a patient underwent a specific treatment or not, and the data available are too incomplete to perform such analysis retrospectively. Still, according to the 2009 ATA Guidelines [38] (the last version is 2015, but it applied only to 1 year worth of patients in the present study), some patients with metastases could be observed without undergoing treatment of the primary lesion.

Other studies have reported a trend that multiple-site metastases were associated with an increased risk of death [22]. Nevertheless, after comparing the survival data of isolated lung metastasis with that of multiple-organ metastasis, including lung metastasis, the prognosis of isolated lung metastasis from TC was like that of lung–bone metastasis from TC. Conversely, there was a significant difference between the prognosis of isolated lung metastasis and other multiple-organ metastasis, including lung metastasis (≥2 organs). The co-occurrence of two metastatic lesions with a relatively good prognosis did not change the prognosis.

In this study, compared to white race patients, other races, except the black race, had a survival advantage in TCSS when lung metastases occurred. The other race contained Hispanics. Avital Harari et al. (2014) reported similar conclusions—that Hispanics had a survival advantage in metastatic TC disease [39]. Referring to other studies [39,40,41], it could not be fully explained by socioeconomic factors and various forms of healthcare insurance. Even more, some studies have reported that the white race has a better prognosis. Since the results of different studies are inconsistent at present, the association between ethnicity and survival has not been established. Rubinstein et al. [42] reported race as an independent prognostic factor of recurrence in TC. From the aspects of the intrinsic differences in tumor biology, genome and/or gene variation were also considered as a possible explanation for the differences between races [43]. In addition, a small study using microRNA expression profiles reported that there were microRNA expression differences between races that might also be related to the potential variation in tumor aggressiveness [44]. 

Because of changes in the diagnostic criteria throughout time, the absence or updating of some key variables, and the impact of selection bias and information bias, the interpretation of the above results should be cautious. Furthermore, prospective controlled studies should be undertaken to evaluate the prognosis of the patients, which could avoid many interference factors and allow further analysis. Due to the low incidence of TC metastasis in the liver, lung, bone, and brain, we could not provide further support from our own biological/cohort data. It must be mentioned that due to the inherent limitations of SEER [45], the impact of adjuvant therapy such as iodine suppression therapy could not be examined on the prognosis. This work would take a long time to accumulate enough cases, and multicenter clinical studies or large-scale databases with more details might help. Finally, the frequency of brain metastases is low. Even in the SEER database, only 87 patients with brain metastases were observed over 7 years, among which 23 were isolated brain metastases, and 64 were brain metastases combined with other distant metastases. The low incidence and small sample size might be the reason why the difference between brain metastases and lung metastases did not differ greatly.

In conclusion, based on the SEER analysis, the lung is the most prevalent site of metastasis from TC. Similar to brain metastasis, the lung metastasis of TC had a worse outcome than the liver or bone metastasis. A worse survival outcome was observed in patients with multiorgan metastases. More research is needed to figure out the exact subset of patients who may benefit from local treatment for primary lesions and/or metastatic sites.

## Figures and Tables

**Table 1:** Clinical features of TC patients with or without lung metastasis from thyroid cancer.

Variable	Lung Metastasis from Thyroid Cancer			*p*
	Yes	No	All	
	(*n* = 998)	(*n* = 76,324)	(*n* = 77,322)	
Age				<0.0001
≥55	249 (24.9%)	50,765 (66.5%)	51,014 (66.0%)	
<55	749 (75.1%)	25,559 (33.5%)	26,308 (34.0%)	
Race				<0.0001
White	745 (74.7%)	60,807 (79.7%)	61,552 (79.6%)	
Black	100 (10.0%)	5413 (7.1%)	5513 (7.1%)	
Unknown	4 (0.4%)	1308 (1.7%)	1312 (1.7%)	
Others *	149 (14.9%)	8796 (11.5%)	8945 (11.6%)	
Sex				<0.0001
Male	458 (45.9%)	17,547 (23.0%)	18,005 (23.3%)	
Female	540 (54.1%)	58,777 (77.0%)	59,317 (76.7%)	
Married				0.5408
Married	4 (0.4%)	177 (0.2%)	181 (0.2%)	
Unmarried	994 (99.6%)	76,145 (99.8%)	77,139 (99.8%)	
Unknown	0	2 (0.0%)	2 (0.0%)	
Grade				<0.0001
I	74 (7.4%)	14,684 (19.2%)	14,758 (19.1%)	
II	39 (3.9%)	2748 (3.6%)	2787 (3.6%)	
III	104 (10.4%)	754 (1.0%)	858 (1.1%)	
VI	295 (29.6%)	492 (0.6%)	787 (1.0%)	
Unknown	486 (48.7%)	57,646 (75.5%)	58,132 (75.2%)	
Histologic ICD-O-3 for thyroid cancer				<0.0001
Carcinoma, undiff., NOS	215 (21.5%)	327 (0.4%)	542 (0.7%)	
Follicular Adenocarcinoma, NOS	99 (9.9%)	3632 (4.8%)	3731 (4.8%)	
Medullary carcinoma, NOS	39 (3.9%)	1186 (1.6%)	1225 (1.6%)	
Oxyphilic adenocarcinoma	28 (2.8%)	1338 (1.8%)	1366 (1.8%)	
Papillary and follicular adenoca.	137 (13.7%)	26,399 (34.6%)	26,536 (34.3%)	
Papillary adenocarcinoma, NOS	312 (31.3%)	40,869 (53.6%)	41,181 (53.3%)	
Papillary carcinoma, NOS	10 (1.0%)	1686 (2.2%)	1696 (2.2%)	
Others	158 (15.8%)	887 (1.2%)	1045 (1.4%)	
Stage group				<0.0001
I	0	53,510 (70.1%)	53,510 (69.2%)	
II	92 (9.2%)	5540 (7.3%)	5632 (7.3%)	
III	0	9761 (12.8%)	9761 (12.6%)	
VI	896 (89.8%)	4989 (6.5%)	5885 (7.6%)	
Unknown	10 (1.0%)	2524 (3.3%)	2534 (3.3%)	
T stage				<0.0001
T0	6 (0.6%)	107 (0.1%)	113 (0.2%)	
T1	47 (4.7%)	43,527 (57.0%)	43,574 (56.4%)	
T2	48 (4.8%)	12,815 (16.8%)	12,863 (16.6%)	
T3	209 (20.9%)	15,575 (20.4%)	15,784 (20.4%)	
T4	560 (56.1%)	2420 (3.2%)	2980 (3.9%)	
Tx	128 (12.8%)	1880 (2.5%)	2008 (2.6%)	
N stage				<0.0001
N0	279 (28.0%)	56,099 (73.5%)	56,378 (72.9%)	
N1	613 (61.4%)	18,044 (23.6%)	18,657 (24.1%)	
Nx	106 (10.6%)	2181 (2.9%)	2287 (3.0%)	
M stage				<0.0001
M0	0	75,590 (99.0%)	75,590 (97.8%)	
M1	988 (99.0%)	603 (0.8%)	1591 (2.1%)	
Mx	10 (1.0%)	131 (0.2%)	141 (0.2%)	
Regional				<0.0001
Positive	407 (40.8%)	17,729 (23.2%)	18,136 (23.5%)	
Negative	75 (7.5%)	23,536 (30.8%)	23,611 (30.5%)	
Unknown	516 (51.7%)	35,059 (45.9%)	35,575 (46.0%)	
SP				<0.0001
Yes	543 (54.4%)	74,221 (97.2%)	74,764 (96.7%)	
No	454 (45.5%)	2067 (2.7%)	2521 (3.3%)	
Unknown	1 (0.1%)	36 (0.1%)	37 (0.1%)	
SD				<0.0001
Yes	118 (11.8%)	1025 (1.3%)	1143 (1.5%)	
No	879 (88.1%)	75,177 (98.5%)	76,056 (98.4%)	
Unknown	1 (0.1%)	122 (0.2%)	123 (0.2%)	

Note: NOS = not otherwise specified; SP = Surgery of the primary; SD = Surgery of other regional disease. * “Other” means any race other than white, black, or unknown.

**Table 2:** Multivariable analyses of overall survival and thyroid cancer-specific survival in TC patients with lung metastasis from thyroid cancer.

Variable		Overall Survival		Thyroid Cancer-Specific Survival	
		Hazard Ratio (95%CI)	*p*	Hazard Ratio (95%CI)	*p*
Age	≥55	1.00 (reference)		1.00 (reference)	
	<55	0.5205 (0.3845–0.7045)	*p* < 0.0001	0.5645 (0.4126–0.7723)	0.0003
Race	White	1.00 (reference)		1.00 (reference)	
	Black	0.8355 (0.5649–1.2355)	0.3678	0.7233 (0.4711–1.1106)	0.1387
	Others	0.7582 (0.5706–1.0073)	0.0562	0.7166 (0.5305–0.9680)	0.0298
Sex	Female	1.00 (reference)		1.00 (reference)	
	Male	0.832 (0.0.6792–1.0191)	0.0756	0.7944 (0.6424–0.9825)	0.0338
Married	Married	1.00 (reference)		1.00 (reference)	
	Unmarried	0.4467 (0.1400–1.4251)	0.1734	0.3828 (0.1198–1.2234)	0.1053
T stage	T1	1.00 (reference)		1.00 (reference)	
	T0	- #	- #	- #	- #
	T2	0.8199 (0.2624–2.5620)	0.7327	0.7737 (0.1919–3.1201)	0.7184
	T3	1.2327 (0.5131–2.9615)	0.6399	1.5424 (0.5375–4.4258)	0.4204
	T4	5.0814 (2.2417–11.5184)	0.0001	7.1042 (2.6239–19.2344)	0.0001
	Tx	2.5526 (1.0826–6.0185)	0.0322	3.3817 (1.2042–9.4965)	0.0207
N stage	N0	1.00 (reference)		1.00 (reference)	
	N1	1.1283 (0.8793–1.4476)	0.3427	1.0394 (0.8023–1.3465)	0.7700
	Nx	1.3251 (0.9408–1.8663)	01072	1.2687 (0.9034–1.8327)	0.1624
SP	No	1.00 (reference)		1.00 (reference)	
	Yes	0.2273 (0.1779–0.2904)	<0.0001	0.2294 (0.1776–0.2963)	<0.0001
SD	No	1.00 (reference)		1.00 (reference)	
	Yes	0.5713 (0.3763–0.8674)	0.0086	0.5302 (0.3371–0.8347)	0.0061

Note: SP = Surgery of the primary; SD = Surgery of other regional disease. # The amount of data is little and the statistical error may be too large. This item will not be analyzed.

## Data Availability

All data generated or analyzed during this study are included in this published article and supplementary materials.

## Supplementary Materials

The following supporting information can be downloaded at: https://www.mdpi.com/article/10.3390/cancers14133133/s1, Figure S1: Kaplan-Meier curves of overall and thyroid cancer-specific survival according to whether surgery or not of the primary thyroid cancer (TC) lesion and distant metastatic lymph nodes (LN) or bone metastasis; Figure S2: Kaplan-Meier curves of overall and thyroid cancer-specific survival according to whether surgery or not of the primary thyroid cancer (TC) lesion and distant metastatic lymph nodes (LN) or liver metastasis; Figure S3: Kaplan-Meier curves of overall and thyroid cancer-specific survival according to whether surgery or not of the primary thyroid cancer (TC) lesion and distant metastatic lymph nodes (LN) or brain metastasis. Table S1: Clinical features of TC patients with or without metastases of other organs.
