# Supplementary material for: Impact of Lung Metastasis versus Metastasis of Bone, Brain, or Liver on Overall Survival and Thyroid Cancer-Specific Survival of Thyroid Cancer Patients: A Population-Based Study
Source: Cancers (Basel). 2022 Jun 26;14(13):3133. doi: 10.3390/cancers14133133 (PMC9265095; doi:10.3390/cancers14133133)
Supplement: Supplementary file 1 [file cancers-14-03133-s001.zip › Supplementary documents (with supplementary Figures).pdf]

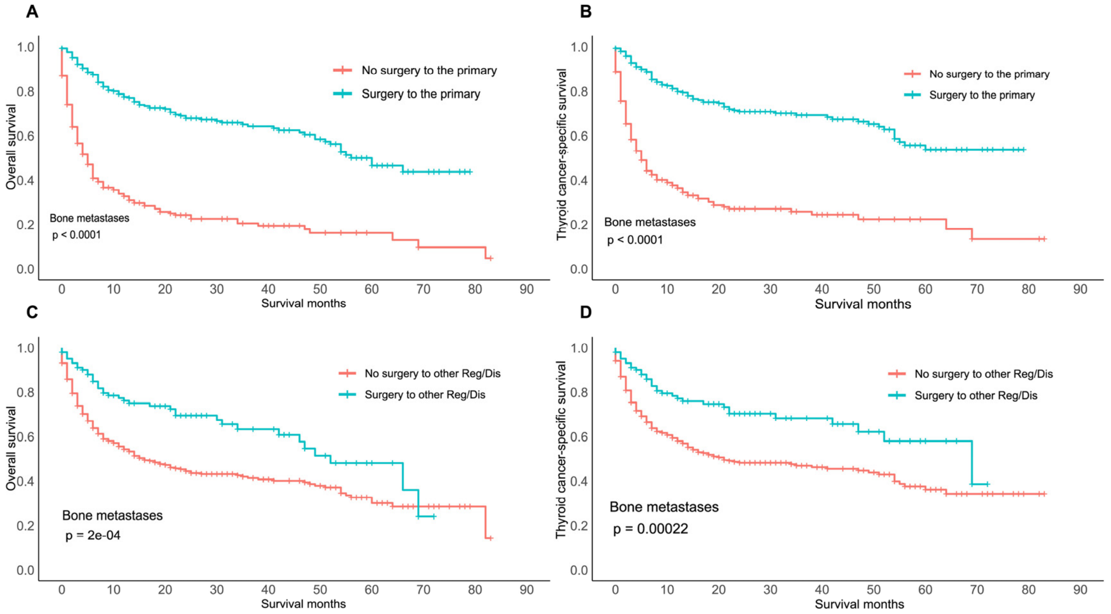

Figure S1. Kaplan-Meier curves of overall and thyroid cancer-specific survival according to whether surgery or not of the primary thyroid cancer (TC) lesion and distant metastatic lymph nodes (LN) or bone metastasis. (A) Overall survival for patients with isolated bone metastasis with or without surgery of the primary tumor. (B) Thyroid cancer-specific survival for patients with isolated bone metastasis with or without surgery of the primary tumor. (C) Overall survival for patients with isolated bone metastasis with or without surgery of the distant lymph nodes (LNs) or other metastatic sites. (D) Thyroid cancer-specific survival for patients with isolated bone metastasis with or without surgery of the distant lymph nodes (LNs) or other metastatic sites.

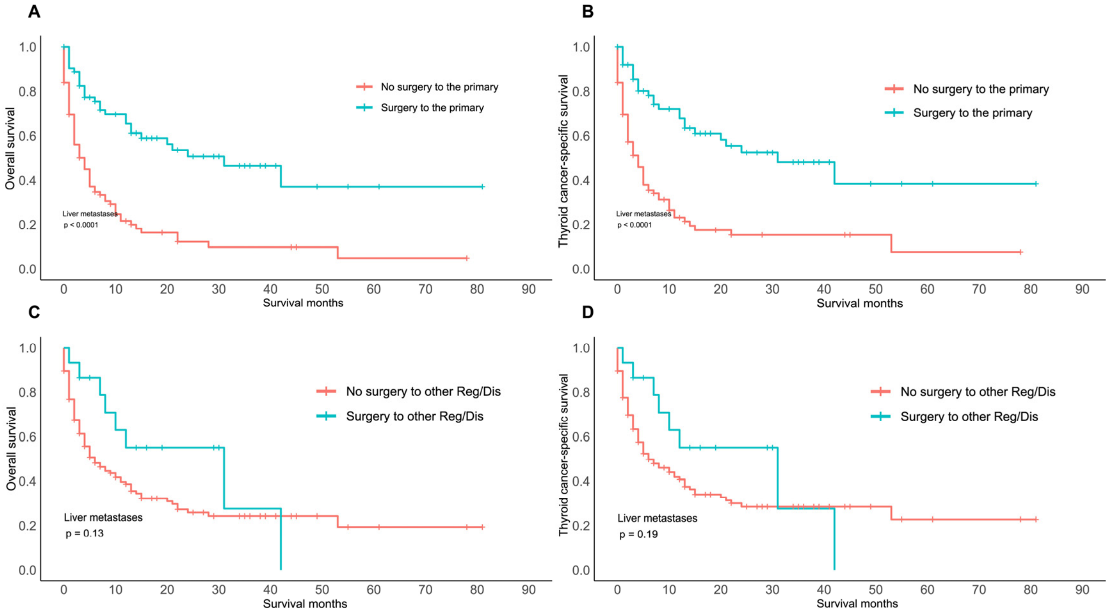

Figure S2. Kaplan-Meier curves of overall and thyroid cancer-specific survival according to whether surgery or not of the primary thyroid cancer (TC) lesion and distant metastatic lymph nodes (LN) or liver metastasis. (A) Overall survival for patients with isolated liver metastases with or without surgery of the primary tumor. (B) Thyroid cancer-specific survival for patients with isolated liver metastases with or without surgery of the primary tumor. (C) Overall survival for patients with isolated liver metastases with or without surgery of the distant lymph nodes (LNs) or other metastatic sites. (D) Thyroid cancer-specific survival for patients with isolated liver metastases with or without surgery of the distant lymph nodes (LNs) or other metastatic sites.

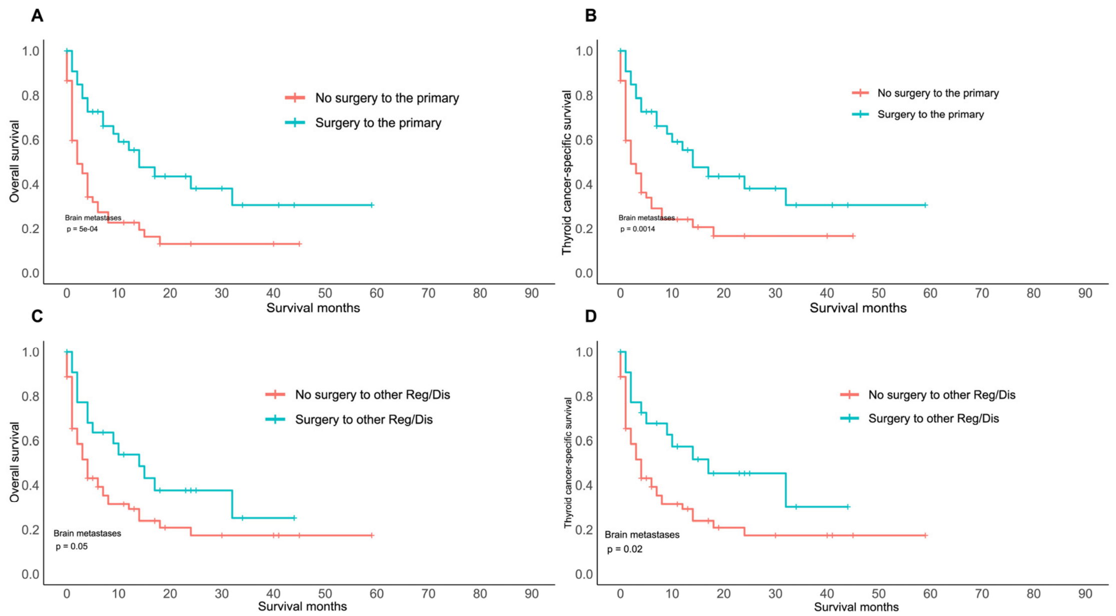

Figure S3. Kaplan-Meier curves of overall and thyroid cancer-specific survival according to whether surgery or not of the primary thyroid cancer (TC) lesion and distant metastatic lymph nodes (LN) or brain metastasis. (A) Overall survival for patients with isolated brain metastases with or without surgery of the primary tumor. (B) Thyroid cancer-specific survival for patients with isolated brain metastases with or without surgery of the primary tumor. (C) Overall survival for patients with isolated brain metastases with or without surgery of the distant lymph nodes (LNs) or other metastatic sites. (D) Thyroid cancer-specific survival for patients with isolated brain metastases with or without surgery of the distant lymph nodes (LNs) or other metastatic sites
