# Supplementary material for: Impact of Lung Metastasis versus Metastasis of Bone, Brain, or Liver on Overall Survival and Thyroid Cancer-Specific Survival of Thyroid Cancer Patients: A Population-Based Study
Source: Cancers (Basel). 2022 Jun 26;14(13):3133. doi: 10.3390/cancers14133133 (PMC9265095; doi:10.3390/cancers14133133)
Supplement: Supplementary file 1 [file cancers-14-03133-s001.zip › Supplementary Tabels.pdf]

Table S1. Clinical features of TC patients with or without metastases of other organs.

Features	**Bone metastases** from thyroid cancer			**P value**	**Liver metastases** from thyroid cancer			**P value**	**Brain metastases** from thyroid cancer			**P value**
	**Yes (N=514)**	**No (N=76808)**	**Total (N=77322)**		**Yes (N=152)**	**No (N=77170)**	**Total (N=77322)**		**Yes (N=87)**	**No (N=77235)**	**Total (N=77322)**	
Age				<0.0001				<0.0001				<0.0001
≥55	128(24.90%)	50886(66.25%)	51014(65.98%)		39(25.66%)	50975(66.06%)	51014(65.98%)		27(31.03%)	50987(66.02%)	51014(65.98%)	
< 55	386(75.10%)	25922(33.75%)	26308(34.02%)		113(74.34%)	26195(33.94%)	26308(34.02%)		60(68.97%)	26248(33.98%)	26308(34.02%)	
Race				<0.0001				0.063				0.0022
White	347(67.51%)	61205(79.69%)	61552(79.60%)		122(80.26%)	61430(79.60%)	61552(79.60%)		60(68.97%)	61492(79.62%)	61552(79.60%)	
Black	80(15.56%)	5433(7.07%)	5513(7.13%)		17(11.18%)	5496(7.12%)	5513(7.13%)		6(6.90%)	5507(7.13%)	5513(7.13%)	
Unknown	0(0.00%)	1312(1.71%)	1312(1.70%)		0(0.00%)	1312(1.70%)	1312(1.70%)		0(0.00%)	1312(1.70%)	1312(1.70%)	
Others*	87(16.93%)	8858(11.53%)	8945(11.57%)		13(8.55%)	8932(11.57%)	8945(11.57%)		21(24.14%)	8924(11.55%)	8945(11.57%)	

Gender				<0.0001				<0.0001				<0.0001
Male	226(43.97%)	17779(23.15%)	18005(23.29%)		69(45.39%)	17936(23.24%)	18005(23.29%)		36(41.38%)	17969(23.27%)	18005(23.29%)	
Female	288(56.03%)	59029(76.85%)	59317(76.71%)		83(54.61%)	59234(76.76%)	59317(76.71%)		51(58.62%)	59266(76.73%)	59317(76.71%)	
Married				0.9763				0.835				0.2094
Married	1(0.19%)	180(0.23%)	181(0.23%)		0(0.00%)	181(0.23%)	181(0.23%)		1(1.15%)	180(0.23%)	181(0.23%)	
Unmarried	513(99.81%)	76626(99.76%)	77139(99.76%)		152(100.00%)	76987(99.76%)	77139(99.76%)		86(98.85%)	77053(99.76%)	77139(99.76%)	
Unknown	0(0.00%)	2(0.00%)	2(0.00%)		0(0.00%)	2(0.00%)	2(0.00%)		0(0.00%)	2(0.00%)	2(0.00%)	
Grade				<0.0001				<0.0001				<0.0001
GradeI	53(10.31%)	14705(19.15%)	14758(19.09%)		3(1.97%)	14755(19.12%)	14758(19.09%)		5(5.75%)	14753(19.10%)	14758(19.09%)	
GradeII	20(3.89%)	2767(3.60%)	2787(3.60%)		2(1.32%)	2785(3.61%)	2787(3.60%)		2(2.30%)	2785(3.61%)	2787(3.60%)	
GradeIII	55(10.70%)	803(1.05%)	858(1.11%)		18(11.84%)	840(1.09%)	858(1.11%)		11(12.64%)	847(1.10%)	858(1.11%)	
GradeVI	87(16.93%)	700(0.91%)	787(1.02%)		33(21.71%)	754(0.98%)	787(1.02%)		29(33.33%)	758(0.98%)	787(1.02%)	
Unknown	299(58.17%)	57833(75.30%)	58132(75.18%)		96(63.16%)	58036(75.21%)	58132(75.18%)		40(45.98%)	58092(75.21%)	58132(75.18%)	

Histologic ICD-O-3 for thyroid cancer				<0.0001				<0.0001				<0.0001
Carcinoma,undiff., NOS	63(12.26%)	479(0.62%)	542(0.70%)		26(17.11%)	516(0.67%)	542(0.70%)		22(25.29%)	520(0.67%)	542(0.70%)	
Follicular adenocarcinoma,NOS	130(25.29%)	3601(4.69%)	3731(4.83%)		10(6.58%)	3721(4.82%)	3731(4.83%)		15(17.24%)	3716(4.81%)	3731(4.83%)	
Medullary carcinoma,NOS	46(8.95%)	1179(1.53%)	1225(1.58%)		53(34.87%)	1172(1.52%)	1225(1.58%)		8(9.20%)	1217(1.58%)	1225(1.58%)	
Oxyphilic adenocarcinoma	22(4.28%)	1344(1.75%)	1366(1.77%)		7(4.61%)	1359(1.76%)	1366(1.77%)		0(0.00%)	1366(1.77%)	1366(1.77%)	
Papillary & follicular adenoca.	100(19.46%)	26436(34.42%)	26536(34.32%)		9(5.92%)	26527(34.37%)	26536(34.32%)		6(6.90%)	26530(34.35%)	26536(34.32%)	
Papillary adenocarcinoma,NOS	95(18.48%)	41086(53.49%)	41181(53.26%)		27(17.76%)	41154(53.33%)	41181(53.26%)		21(24.14%)	41160(53.29%)	41181(53.26%)	
Papillary carcinoma,NOS	5(0.97%)	1691(2.20%)	1696(2.19%)		0(0.00%)	1696(2.20%)	1696(2.19%)		0(0.00%)	1696(2.20%)	1696(2.19%)	
Others	53(10.31%)	992(1.29%)	1045(1.35%)		20(13.16%)	1025(1.33%)	1045(1.35%)		15(17.24%)	1030(1.33%)	1045(1.35%)	
Stage group				<0.0001				<0.0001				<0.0001

I	0(0.00%)	53510(69.67%)	53510(69.20%)		0(0.00%)	53510(69.34%)	53510(69.20%)		0(0.00%)	53510(69.28%)	53510(69.20%)	
II	28(5.45%)	5604(7.30%)	5632(7.28%)		3(1.97%)	5629(7.29%)	5632(7.28%)		7(8.05%)	5625(7.28%)	5632(7.28%)	
III	0(0.00%)	9761(12.71%)	9761(12.62%)		0(0.00%)	9761(12.65%)	9761(12.62%)		0(0.00%)	9761(12.64%)	9761(12.62%)	
VI	483(93.97%)	5402(7.03%)	5885(7.61%)		148(97.37%)	5737(7.43%)	5885(7.61%)		80(91.95%)	5805(7.52%)	5885(7.61%)	
Unknown	3(0.58%)	2531(3.30%)	2534(3.28%)		1(0.66%)	2533(3.28%)	2534(3.28%)		0(0.00%)	2534(3.28%)	2534(3.28%)	
T				<0.0001				<0.0001				<0.0001
T0	10(1.95%)	103(0.13%)	113(0.15%)		0(0.00%)	113(0.15%)	113(0.15%)		1(1.15%)	112(0.15%)	113(0.15%)	
T1	50(9.73%)	43524(56.67%)	43574(56.35%)		8(5.26%)	43566(56.45%)	43574(56.35%)		6(6.90%)	43568(56.41%)	43574(56.35%)	
T2	58(11.28%)	12805(16.67%)	12863(16.64%)		11(7.24%)	12852(16.65%)	12863(16.64%)		5(5.75%)	12858(16.65%)	12863(16.64%)	
T3	117(22.76%)	15667(20.40%)	15784(20.41%)		32(21.05%)	15752(20.41%)	15784(20.41%)		17(19.54%)	15767(20.41%)	15784(20.41%)	
T4	193(37.55%)	2787(3.63%)	2980(3.85%)		69(45.39%)	2911(3.77%)	2980(3.85%)		46(52.87%)	2934(3.80%)	2980(3.85%)	
Tx	86(16.73%)	1922(2.50%)	2008(2.60%)		32(21.05%)	1976(2.56%)	2008(2.60%)		12(13.79%)	1996(2.58%)	2008(2.60%)	
N												

				<0.0001				<0.0001				<0.0001
N0	233(45.33%)	56145(73.10%)	56378(72.91%)		40(26.32%)	56338(73.01%)	56378(72.91%)		31(35.63%)	56347(72.96%)	56378(72.91%)	
N1	221(43.00%)	18436(24.00%)	18657(24.13%)		99(65.13%)	18558(24.05%)	18657(24.13%)		42(48.28%)	18615(24.10%)	18657(24.13%)	
Nx	60(11.67%)	2227(2.90%)	2287(2.96%)		13(8.55%)	2274(2.95%)	2287(2.96%)		14(16.09%)	2273(2.94%)	2287(2.96%)	
M				<0.0001				<0.0001				<0.0001
M0	0(0.00%)	75590(98.41%)	75590(97.76%)		0(0.00%)	75590(97.95%)	75590(97.76%)		0(0.00%)	75590(97.87%)	75590(97.76%)	
M1	511(99.42%)	1080(1.41%)	1591(2.06%)		151(99.34%)	1440(1.87%)	1591(2.06%)		87(100.00%)	1504(1.95%)	1591(2.06%)	
Mx	3(0.58%)	138(0.18%)	141(0.18%)		1(0.66%)	140(0.18%)	141(0.18%)		0(0.00%)	141(0.18%)	141(0.18%)	
Regional				<0.0001				<0.0001				0.0006
Positive	144(28.02%)	17992(23.42%)	18136(23.46%)		58(38.16%)	18078(23.43%)	18136(23.46%)		26(29.89%)	18110(23.45%)	18136(23.46%)	
Negtive	75(14.59%)	23536(30.64%)	23611(30.54%)		8(5.26%)	23603(30.59%)	23611(30.54%)		10(11.49%)	23601(30.56%)	23611(30.54%)	

	Unknown	295(57.39%)	35280(45.93%)	35575(46.01%)		86(56.58%)	35489(45.99%)	35575(46.01%)		51(58.62%)	35524(45.99%)	35575(46.01%)	
SP					<0.0001				<0.0001				<0.0001
	Yes	298(57.98%)	74466(96.95%)	74764(96.69%)		64(42.11%)	74700(96.80%)	74764(96.69%)		35(40.23%)	74729(96.76%)	74764(96.69%)	
	No	216(42.02%)	2305(3.00%)	2521(3.26%)		87(57.24%)	2434(3.15%)	2521(3.26%)		52(59.77%)	2469(3.20%)	2521(3.26%)	
	Unknown	0(0.00%)	37(0.05%)	37(0.05%)		1(0.66%)	36(0.05%)	37(0.05%)		0(0.00%)	37(0.05%)	37(0.05%)	
SD					<0.0001				<0.0001				<0.0001
	Yes	103(20.04%)	1040(1.35%)	1143(1.48%)		15(9.87%)	1128(1.46%)	1143(1.48%)		24(27.59%)	1119(1.45%)	1143(1.48%)	
	No	410(79.77%)	75646(98.49%)	76056(98.36%)		136(89.47%)	75920(98.38%)	76056(98.36%)		63(72.41%)	75993(98.39%)	76056(98.36%)	
	Unknown	1(0.19%)	122(0.16%)	123(0.16%)		1(0.66%)	122(0.16%)	123(0.16%)		0(0.00%)	123(0.16%)	123(0.16%)	

Note: NOS = not otherwise specified; SP = Surgery of the primary; SD = Surgery of other regional disease

*"Other" means any race other than white, black, or unknown.
